# Quality of Life, Anxiety and Depression in Women Treated with Hysteroscopic Endometrial Resection or Ablation for Heavy Menstrual Bleeding: Systematic Review and Meta-Analysis of Randomized Controlled Trials

**DOI:** 10.3390/medicina58111664

**Published:** 2022-11-17

**Authors:** Salvatore Giovanni Vitale, Gaetano Riemma, Mislav Mikuš, Jose Carugno, Marco Torella, Enrique Reyes-Muñoz, Vito Cela, Tirso Perez Medina, Luigi Della Corte, Luis Alonso Pacheco, Sergio Haimovich, Pasquale De Franciscis, Stefano Angioni

**Affiliations:** 1 Division of Gynecology and Obstetrics, Department of Surgical Sciences, University of Cagliari, 09124 Cagliari, Italy; 2 Obstetrics and Gynecology Unit, Department of Woman, Child and General and Specialized Surgery, University of Campania “Luigi Vanvitelli”, 80128 Naples, Italy; 3 Department of Obstetrics and Gynecology, University Hospital Center Zagreb, 10000 Zagreb, Croatia; 4 Obstetrics, Gynecology and Reproductive Sciences Department, Minimally Invasive Gynecology Unit, University of Miami, Miller School of Medicine, Miami, FL 33124, USA; 5 Department of Gynecological and Perinatal Endocrinology, Instituto Nacional de Perinatología, Mexico City 11000, Mexico; 6 Division of Gynecology and Obstetrics, Department of Clinical and Experimental Medicine, University of Pisa, 56126 Pisa, Italy; 7 Department of Obstetrics and Gynecology, University Hospital Puerta de Hierro Majadahonda, Autonoma University of Madrid, 28001 Madrid, Spain; 8 Department of Neuroscience, Reproductive Sciences and Dentistry, School of Medicine, University of Naples Federico II, 80131 Naples, Italy; 9 Unidad de Endoscopia Ginecológica, Centro Gutenberg, 29010 Málaga, Spain; 10 Department of Obstetrics and Gynecology, Laniado University Hospital, Netanya, Israel and Adelson School of Medicine, Ariel University, Ariel 98603, Israel

**Keywords:** endometrial ablation, hysteroscopy, hysterectomy, heavy menstrual bleeding, abnormal uterine bleeding, metrorrhagia

## Abstract

*Background and Objectives*: Hysteroscopic endometrial resection (ER) or global endometrial ablation (GEA) are feasible methods to treat heavy menstrual bleeding (HMB). The aim of this systematic review and meta-analysis of randomized controlled trials (RCTs) was to assess patient’s quality of life (QoL) in women treated with ER/GEA compared to hysterectomy. *Materials and Methods*: Electronic searches in MEDLINE Scopus, ClinicalTrials.gov, EMBASE, PROSPERO and Cochrane CENTRAL were conducted from their inception to July 2022. Inclusion criteria were RCTs of premenopausal women with HMB randomized to conservative surgical treatment (ER/GEA) or hysterectomy. The primary outcome was the evaluation of QoL using the SF-36 score. *Results*: Twelve RCTs (2773 women) were included in the analysis. Women treated with hysteroscopic ER/GEA showed significantly lower scores for the SF-36 general health perception (mean difference (MD) −8.56 [95% CI −11.75 to −5.36]; I^2^ = 0%), social function (MD −12.90 [95% CI −23.90 to −1.68]; I^2^ = 91%), emotional role limitation (MD −4.64 [95% CI −8.43 to −0.85]; I^2^ = 0%) and vitality (MD −8.01 [95% CI −14.73 to −1.30]; I^2^ = 74%) domains relative to hysterectomy. Anxiety, depression scores and complication rates were similar between treatments. Relative to uterine balloon therapy, amenorrhea was more common with EA/GER (relative risk 1.51 [95% CI 1.03 to 1.20] I^2^ = *28*%), but posttreatment satisfaction was similar. *Conclusions*: Women’s perception of QoL might be seen to be less improved after hysteroscopic ER/GEA rather than hysterectomy. However, such findings need to be confirmed by additional trials due to the high number of outdated studies and recent improvements in hysteroscopic instrumentation and techniques.

## 1. Introduction

In recent decades, the development of minimally invasive procedures has led to the expansion of the range of operations in cases where traditional methods are not applicable [1,2,3]. In gynecologic practice, hysteroscopy has become the gold standard for the evaluation and treatment of intracavitary pathology. In fact, operative hysteroscopy (OH) represents the treatment of choice for endocavitary lesions such as polyps, fibroids and endometrial anomalies [4,5,6]. Heavy menstrual bleeding (HMB) is one of the most common reasons for referral to gynecologic services. It affects up to 30% of women of reproductive age and is one of the most important symptoms of premalignant/malignant lesions in postmenopausal women [7,8,9,10,11]. Although the medical approach is the first choice, hysteroscopic instruments can not only help in the diagnostic orientation by excluding pathologies such as endometrial polyps, fibroids, endometrial dysplasia or intraepithelial neoplasia (EIN) [12,13,14,15,16], but also in the implementation of definitive treatment. In this regard, after the exclusion of neoplasms [17], hysteroscopic devices such as rollerball electrodes, special diathermal slings, hysteroscopic morcellators or endocavitary ablation devices such as the uterine balloon therapy (UBT) have been developed to destroy the endometrial layer partially or completely [18,19,20] aiming to decrease/eliminate menstrual blood flow. Endometrial resection (ER) using a rollerball or a bipolar loop can reduce and even abolish HMB in reproductive-aged and perimenopausal women. When ablation encompasses the entire endometrial cavity and the entire endometrium is removed down to the basalis layer, it is referred to as endometrial ablation (EA) [21]. EA is currently used as an alternative minimally invasive technique in cases where hysterectomy wants to be avoided, and medical treatments are inconsistent. EA offers an alternative to hysterectomy as a surgical treatment for HMB. Both procedures are effective, and satisfaction rates are high. Although hysterectomy offers permanent and immediate relief from HMB, it is associated with a higher cost, and longer operating time and recovery period, as well as higher rates of postoperative complications such as sepsis, blood transfusion and hematoma [9]. However, despite it being established that hysteroscopic or instrumental ablation of the endometrium has a similar efficacy to hysterectomy, it is still less considered by patients. The aim of this systematic review and meta-analysis of randomized controlled trials (RCTs) is to evaluate the impact of hysteroscopic GEA or ER in terms of patient’s perception and postsurgical quality of life (QoL) compared to hysterectomy.

## 2. Materials and Methods

Our meta-analysis followed the Preferred Reporting Item for Systematic Reviews and Meta-analyses (PRISMA) statement [22]. The research protocol was designed a priori, and it carefully addressed the literature search and reporting, inclusion and analysis of articles, data extraction and statistical analysis.

### 2.1. Data Sources

We conducted an electronic research in MEDLINE (accessed through PubMed), Scopus, ClinicalTrials.gov, EMBASE, the PROSPERO International Prospective Register of Systematic Reviews and the Cochrane Central Register of Controlled Trials, performed with the use of the following keywords and Medical Subject Headings (MeSH): “endometrial resection,” “endometrial ablation,” “hysteroscopy” or “resectoscopy,” or “hysterectomy” (Table S1) from inception of each database to July 2022. All results were then limited to “clinical trial.” No language restriction was applied. We did not use any restriction for geographic location. Commentaries, letters to editor, editorials and reviews were excluded from the search in every database. When needed, unpublished data were captured by involving a direct contact with the authors of the original trials whenever the study methodology indicated that further outcome data could have been recorded.

### 2.2. Eligibility Criteria and Data Collection

Inclusion criteria were the following: RCTs of premenopausal women with HMB or AUB unresponsive to medical treatment requesting conservative surgical treatment of their condition. Women were excluded in cases of suspected or confirmed endometrial premalignancy or malignancy, active pelvic infection, submucous myomas, endometrial or cervical polyps, uterine malformation, previous ER or GEA.

The abstraction forms were designed specifically for this review. Key characteristics recorded included: patient descriptors, setting, features of the treatment and comparator, outcomes evaluation, study duration, mean follow-up, results and quality elements.

The abstracts were all evaluated and categorized separately by two authors (G.R. and S.G.V.). Consensus was achieved on possible relevance; the same two authors assessed the complete texts of the chosen studies and separately extracted pertinent information about the study’s features and available results. The reviewers evaluated all the discrepancies, and after consulting a third author, an agreement was established (P.D.F.). When relevant information was not reported, authors were contacted personally in the case of the study methodology suggesting that relevant information may have been captured.

### 2.3. Risk of Bias

The risk of bias in each of the studies included in this analysis was assessed through the criteria described in the Cochrane Handbook for Systematic Reviews of Interventions. Review authors’ judgments were classified as “low risk,” “high risk” or “unclear risk” of bias [23].

### 2.4. Main Outcome Measures

An intention-to-treat approach was used for the analysis. Primary outcome for this review was the patient’s postoperative QoL evaluated using the 36-Item Short Form Health Survey (SF-36) domains. The SF-36 is a collection of general, well-designed and simple to use quality-of-life measures. Clinicians now frequently use these measures, which rely on patient self-reporting, for routine monitoring and assessment of care outcomes in adult patients [24].

Secondary outcomes included the assessment of the following items: complete satisfaction, defined as the rate of women who reported the highest score in the postsurgical satisfaction survey, postoperative anxiety and depression by means of the Hospital Anxiety and Depression Scale (HADS) [25], and the rate of surgical complications among the different techniques.

### 2.5. Data Analysis

The data analysis was carried out using Review Manager 5.3 (The Nordic Cochrane Centre 2014, Copenhagen, Denmark) and STATA 14.1 (StataCorp., College Station, TX, USA). The summary measures were reported as summary mean difference (MD) or risk ratio (RR) with 95% of confidence interval (CI) using the random effects model of Der Simonian and Laird. I-squared (Higgins I^2^) greater than 0% was used to identify potential heterogeneity. The potential publication bias was assessed using the Egger test and visual analysis of funnel plots by two authors (M.M. and S.A.). A *p*-value < 0.05 was considered statistically significant.

## 3. Results

### 3.1. Study Characteristics

**Figure 1:**
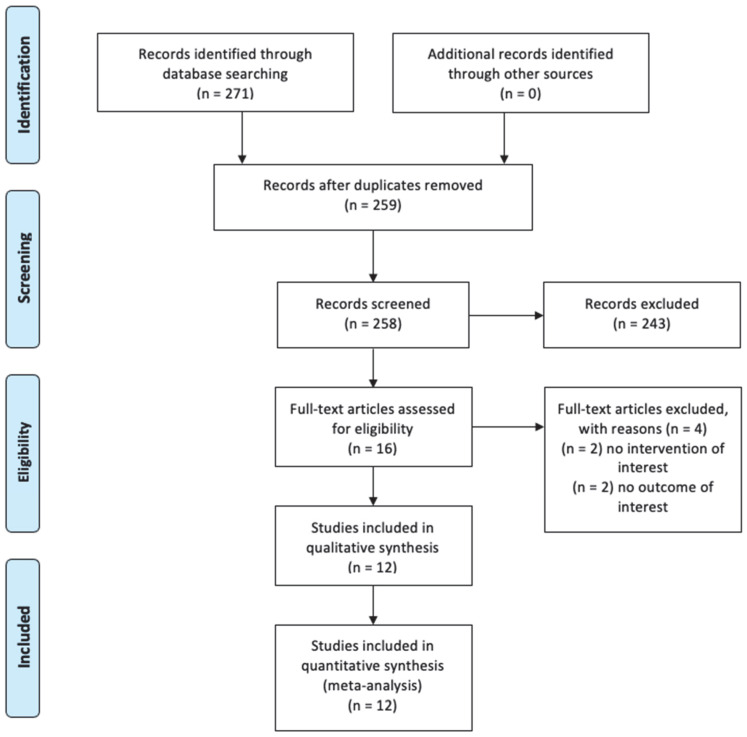
Flow diagram of search strategy according to PRISMA statement.

The original search strategy captured 271 trials, and of those, 12 were removed as duplicates. After title and abstract screening, 243 trials were removed, and 16 papers were selected for full text assessment. Of those, two were removed due to the absence of the investigated intervention and two for the absence of an outcome of interest. Therefore, 12 RCTs, with data provided for 2773 women, were included in the quantitative synthesis and meta-analysis [26,27,28,29,30,31,32,33,34,35,36,37] (Figure 1).

All the studies were RCTs and had a single blind design; two were multicentric, while 9 were single center trials. Hysteroscopic ER or GEA were performed in each trial by means of a 26 Fr continuous flow bipolar resectoscope equipped with a rollerball electrode in an operating room setting with patients under spinal or conscious sedation anesthesia. In the case of laparoscopic or laparotomic hysterectomy, general anesthesia was utilized. The main characteristics of the included RCTs are summarized in Table 1.

Studies were conducted in high-income countries (the United States, England, Scotland, France and Italy) and the trials’ duration ranged from 12 to 34 months. In terms of QoL, depression and anxiety scores, eight RCTs compared resectoscopic ER or GEA to hysterectomy [27,28,30,31,33,34,36,37]. In three papers, the control procedures were laparoscopic supracervical hysterectomies [33,34,37], one trial compared ER/GEA to abdominal hysterectomy [31], one to vaginal hysterectomy [30], while the hysterectomy approach was unspecified in three studies [27,28,36] (Table 1). Three RCTs evaluated the postsurgical satisfaction of ER/GEA relative to UBT ablation [29,32,35] (Table 1).

### 3.2. Quality Assessment

**Figure 2:**
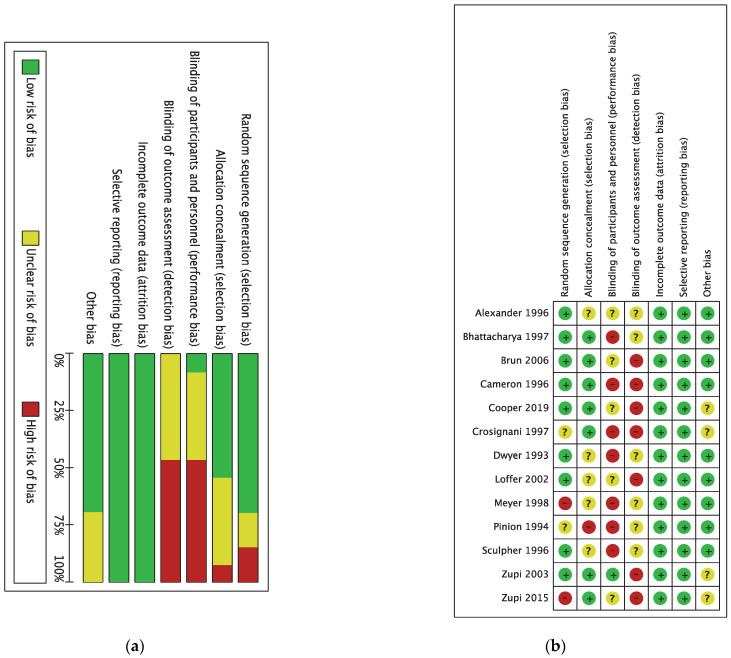
Risk of bias in included RCTs according to Cochrane criteria. (**a**) Summary graph. (**b**) Detailed study-by-study assessment. Plus sign: low risk of bias; minus sign: high risk of bias; question mark: unclear risk of bias.

According to the criteria outlined in the Cochrane Handbook for Systematic Reviews of Interventions, the overall risk of bias was estimated as intermediate by the two authors. Most studies granted a low risk of bias in selective reporting and incomplete outcome data. All the included trials were single blind randomized, with unclear or high bias judgment for performance and detection biases due to unreported or unclear data about blinding of women or personnel. Conversely, selection bias was estimated as low (Figure 2a,b).

### 3.3. EA/ER vs. Hysterectomy

#### 3.3.1. QoL

QoL was evaluated by three studies, providing data for 443 participants. Women subjected to ER/GEA reported significantly lower scores for the general health perception (MD −8.56 [95% CI −11.75 to −5.36]; I^2^ = 0%), social function (MD −12.90 [95% CI −23.90 to −1.68]; I^2^ = 91%), emotional role limitation (MD −4.64 [95% CI −8.43 to −0.85]; I^2^ = 0%) and vitality (MD −8.01 [95% CI −14.73 to −1.30]; I^2^ = 74%).

**Figure 3:**
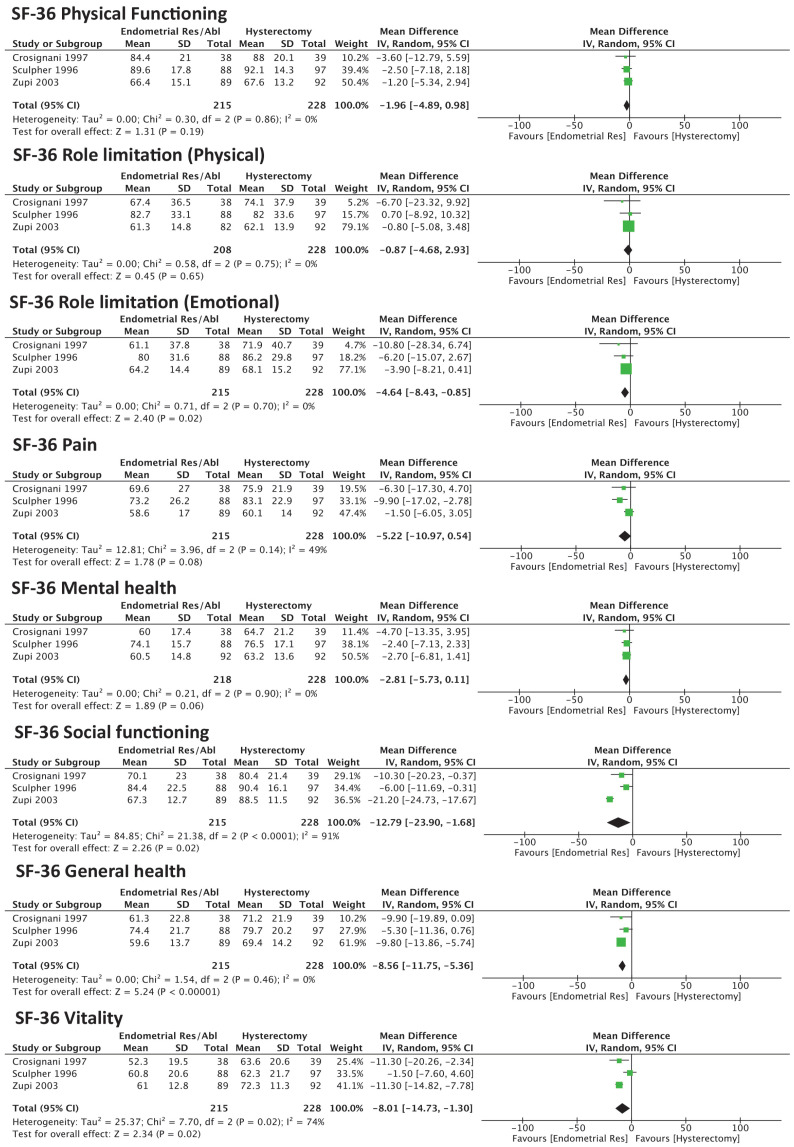
Forest plot for the primary outcome (QoL) evaluated using SF-36 score.

There were no differences regarding the physical functioning, pain and mental health domains (Figure 3).

#### 3.3.2. Anxiety and Depression

**Figure 4:**
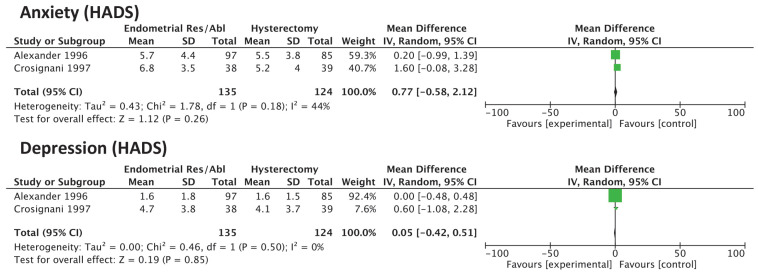
Forest plot for anxiety and depression according to HADS score.

Two RCTs (with 259 women) evaluated changes in anxiety and depression by means of the HADS scale. There were no significant differences for both anxiety and depression for women undergoing EA/GER relative to hysterectomy (Figure 4).

#### 3.3.3. Postsurgical Complications

**Figure 5:**
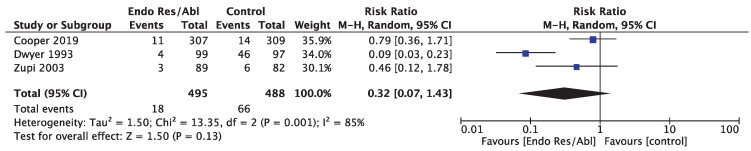
Forest plot for postsurgical complications rates.

The rate of complications after EA/GER or hysterectomy was reported in three studies, providing data for 983 women. No difference in the risk ratio among the two procedures was reported (RR 0.32 [95% CI 0.07 to 1.43]; I^2^ = 85%) (Figure 5).

### 3.4. EA/ER vs. UBT

Three trials compared EA/GER to UBT relative to the amenorrhea rate and four to satisfaction rates. Complete amenorrhea was achieved in a significantly higher number of patients undergoing EA/GER compared to UBT (RR 1.51 [95% CI 1.03 to 1.20] I^2^ = 28%).

Similarly, the number of women who reported high satisfaction for the undergone procedure was similar between the treatments (RR 1.04 [95% CI 0.97 to 1.11] I^2^ = 10%).

## 4. Discussion

This quantitative synthesis and meta-analysis of RCTs revealed that, for five out of eight domains of the SF-36 score, the patient’s perception of noninvasive ER/GEA reported lower scores in comparison to hysterectomy. The postoperative rates of anxiety and depression were similar. Relative to UBT, more women achieved complete amenorrhea but showed comparable satisfaction with the procedure.

EA is indicated in premenopausal women with AUB or HMB of benign etiology after the exclusion of premalignant or malignant conditions. This minimally invasive approach has gained approval among physicians to the point that NICE recommends it as an alternative for women with AUB without uterine abnormalities and with small uterine fibroids (less than 3 cm in diameter) [37,38]. In addition, others have suggested that endometrial ablation is preferable to hysterectomy for women with AUB whose uteruses are not larger than 10 weeks in size [39]. After treatment, amenorrhea is achieved in 14–70% of cases [40,41]. The most common complications are bleeding and uterine perforation, which are easily managed with simple interventions [42]. The need for additional surgery due to perioperative complications is rare and occurs in 1.3% of patients [42]. One of the most reasonable concerns in avoiding the use of GEA or ER instead of a hysterectomy is the risk of endometrial malignancy. As more procedures have been performed in recent decades, incidental findings of premalignant lesions after the procedure have been more frequently found in the literature [43,44,45,46]. These facts raise concerns. On the one hand, cancerous lesions are a contraindication to the procedure; on the other hand, some recent findings seem to demonstrate a similar risk of endometrial cancer between women presenting with AUB/HMB and those who do not. Therefore, the management of these women could be a thought-provoking issue that opens new research scenarios.

To date, there are several options to treat HMB in women with or without uterine fibroids. Recently, ultrasound- and MR-guided high-intensity focused ultrasound (HIFU) has been proposed as an alternative to the surgical removal of fibroids. Combined data showed that HIFU ablation seems superior to conservative surgery in terms of symptomatic relief, improvement in QoL, recovery and significant complications. However, there might be no superiority in terms of reintervention and symptom recurrence [47].

Another minimally invasive approach is based on the use of sonography-guided radiofrequency ablation of uterine fibroids, combining the efficacy of radiofrequency ablation with real-time imaging to improve safety [48]. Recent data showed a marked improvement in menstrual pattern, QoL and fibroid volume. However, the lack of comparative studies with major surgery or other minimally invasive techniques is a limitation to the actual findings [48].

Similarly, uterine artery embolization has shown improvements in QoL, symptoms relief and uterine volume in patients with HMB and uterine fibroids. However, long-term data from a recent multicentric RCT comparing embolization to a standard excisional approach reported that women who underwent myomectomy had a significant QoL improvement 2 years after the procedure relative to those randomized to uterine artery embolization [49]. Therefore, the need for high-quality randomized studies comparing the available approaches still remains.

We acknowledge several limitations of our study that must be considered. First, although included trials were all randomized, there was no double blinding in any study, leading to a higher risk of bias. However, due to the completeness of the reported outcomes and high-quality scores in the other items, the overall risk of bias was reported as intermediate. Another limitation to be acknowledged stands in the hysteroscopic techniques used for GEA/ER, which included the use of the rollerball electrode in all the RCTs. To date, this instrument has been replaced by the loop electrode, with improved clinical outcomes [50,51]. However, there are no studies evaluating the impact of the use of a loop electrode instead of the rollerball on QoL, anxiety and depression. For this reason, it is imperative to perform new trials in lights of the advances in the conservative approach, which is now more frequently requested by women.

Both hysteroscopic procedures and hysterectomy improve QoL when used as a therapeutic option for AUB/HMB; however, two [30,36] of the three RCTs included in the meta-analysis of QoL did not have a baseline measurement.

It is probable that the lower punctuation in the SF-36 score found in the noninvasive ER/GEA studies is related to the repeat surgery or by the years of discomfort before repeat surgery that occur in 12 to 28% of women who were treated with rollerball [34,36].

Nonetheless, this meta-analysis has several points of strength; firstly, the selection of only RCTs, avoiding quasi or nonrandomized studies, to improve the robustness of the findings. Secondly, the heterogeneity between studies was categorized as low for the primary outcome, elevating the quality of the available evidence.

## 5. Conclusions

This quantitative synthesis and meta-analysis of RCTs showed that the patient’s perception of noninvasive ER/GEA, relative to hysterectomy, is better reported for hysterectomy, except for the general health perception, social function and emotional role limitation domains of the SF-36 score. Postoperative anxiety and depression are similar between ER/GEA and hysterectomy. Compared to UBT, complete amenorrhea was achieved in more patients, while a similar treatment satisfaction was reported. However, these findings need to be reassessed with additional trials due to the innovations in the hysteroscopic approach and other minimally invasive equipment available for GEA and ER.

## Figures and Tables

**Table 1:** Main characteristic of studies included in quantitative synthesis and meta-analysis.

Study, Year	Single Center/Multicentric	Design, Blinding	Duration	Location	Hysteroscopic Surgery	Intervention 2	Intervention 3	SS Hysteroscopy	SS Intervention 2	SS Intervention 3	Primary Outcome	Secondary Outcomes
Pinion, 1994 [27]	Single center	RCT, single	12 months	Scotland	26Fr resectoscopic endometrial resection	Laser Ablation	Hysterectomy (unspecified)	52	53	99	Complications	Procedure time
Alexander, 1996 [28]	Single center	RCT, single	12 months	Scotland	26Fr resectoscopic endometrial resection	Laser ablation	Hysterectomy (unspecified)	52	53	99	Anxiety and depression score (HADS)	NA
Bhattacharya, 1997 [26]	Single center	RCT, single	12 months	Scotland	26Fr resectoscopic endometrial resection	Laser ablation	/	184	188	/	Procedure time, fluid overload rate	Amenorrhea rate, 2nd look rate, anxiety and depression score (HADS), dysmenorrhea rate
Brun, 2006 [29]	Multicentric	RCT, single	22 months	France	26Fr resectoscopic endometrial resection	Uterine balloon therapy	/	20	31	/	menorrhea rate and the amount of uterine bleeding.	Satisfaction rate
Crosignani, 1997 [30]	Single center	RCT, single	24 months	Italy	26Fr resectoscopic endometrial resection	Vaginal hysterectomy	/	41	44	/	Anxiety and depression state (HADS)	Sexual function, SF-36
Dwyer, 1993 [31]	Single center	RCT, single	NA	England	26Fr resectoscopic endometrial resection	Abdominal hysterectomy	/	100	100	/	Patient satisfaction, complications; length of hospital stay; change in premenstrual symptoms.	NA
Loffer, 2002 [32]	Single center	RCT, single	12 months	USA	26Fr resectoscopic endometrial resection	Uterine balloon therapy	/	131	124	/	Need for hysterectomy,	Bleeding, satisfaction rate
Zupi, 2003 [33]	Single center	RCT, single	24 months	Italy	26Fr resectoscopic endometrial resection	Laparoscopic supracervical hysterectomy	/	89	92	/	SF-36, operative time	Postoperative course
Zupi, 2015 [34]	Single center	RCT, single	24 months	Italy	26Fr resectoscopic endometrial resection	Laparoscopic supracervical hysterectomy	/	89	92	/	Reintervention rate	SF-36
Sculpher, 1996 [36]	Single center	RCT, single	NA	England	26Fr resectoscopic endometrial resection	Hysterectomy (unspecified)	/	88	97	/	SF-36, operative time	Symptoms, complications, cost-analysis
Cooper, 2019 [37]	Multicentric	RCT, single	34 months	UK	26Fr resectoscopic endometrial resection	Laparoscopic supracervical hysterectomy	/	307	309	/	Patient satisfaction, quality of life (MMAS scale)	Pain, symptoms, complications
Meyer, 1998 [35]	Multicentric	RCT, single	12 months	USA	26Fr resectoscopic endometrial resection	Uterine balloon therapy	/	117	128		Menstrual pattern improvement	Satisfaction, Dysmenorrhea

RCT: randomized controlled trial; SS: sample size; Fr: French; NA: not available.

## Data Availability

The data presented in this study are available on request from the corresponding author.

## Supplementary Materials

The following supporting information can be downloaded at: https://www.mdpi.com/article/10.3390/medicina58111664/s1, Table S1: Search strategy for MEDLINE (accessed through PubMed).

## References

[1] Daniilidis A., Pantelis A., Dinas K., Tantanasis T., Loufopoulos P.D., Angioni S., Carcea F. (2011). Indications of diagnostic hysteroscopy, a brief review of the literature. Gynecol. Surg..

[2] Vitale S.G., Carugno J., Riemma G., Török P., Cianci S., De Franciscis P., Parry J.P. (2020). Hysteroscopy for Assessing Fallopian Tubal Obstruction: A Systematic Review and Diagnostic Test Accuracy Meta-analysis. J. Minim. Invasive Gynecol..

[3] Vitale S.G., Sardo A.D.S., Riemma G., De Franciscis P., Pacheco L.A., Carugno J. (2022). In-office hysteroscopic removal of retained or fragmented intrauterine device without anesthesia: A cross-sectional analysis of an international survey. Updat. Surg..

[4] Luerti M., Vitagliano A., Sardo A.D.S., Angioni S., Garuti G., De Angelis C., Del Zoppo S., Dealberti D., Nappi L., Perrini G. (2019). Effectiveness of Hysteroscopic Techniques for Endometrial Polyp Removal: The Italian Multicenter Trial. J. Minim. Invasive Gynecol..

[5] Vitale S.G., Parry J.P., Carugno J., Cholkeri-Singh A., Della Corte L., Cianci S., Schiattarella A., Riemma G., De Franciscis P. (2020). Surgical and Reproductive Outcomes after Hysteroscopic Removal of Retained Products of Conception: A Systematic Review and Meta-analysis. J. Minim. Invasive Gynecol..

[6] Vitale S.G., Riemma G., Carugno J., Perez-Medina T., Pacheco L.A., Haimovich S., Parry J.P., Sardo A.D.S., De Franciscis P. (2021). Postsurgical barrier strategies to avoid the recurrence of intrauterine adhesion formation after hysteroscopic adhesiolysis: A network meta-analysis of randomized controlled trials. Am. J. Obstet. Gynecol..

[7] Pluchino N., Ninni F., Angioni S., Artini P.G., Araujo V.G., Massimetti G., Genazzani A., Cela V. (2010). Office Vaginoscopic Hysteroscopy in Infertile Women: Effects of Gynecologist Experience, Instrument Size, and Distention Medium on Patient Discomfort. J. Minim. Invasive Gynecol..

[8] Clark T.J. (2004). Outpatient hysteroscopy and ultrasonography in the management of endometrial disease. Curr. Opin. Obstet. Gynecol..

[9] Helleland L., Bergesen L.F., Rinnan K.J., Engelsen I.B., Hordnes K., Trovik J. (2019). Endometrial ablation; less is more? Historical cohort study comparing long-term outcomes from two time periods and two treatment modalities for 854 women. PLoS ONE.

[10] van den Brink M.J., Saaltink A.L., Groenhof F., Kollen B.J., Berger M.Y., Lisman-van Leeuwen Y., Dekker J.H. (2017). Incidence and treatment of heavy menstrual bleeding in general practice. Fam. Pract..

[11] Beelen P., Brink M.J.V.D., Herman M.C., Geomini P.M., Dekker J., Duijnhoven R.G., Mak N., van Meurs H.S., Coppus S.F., van der Steeg J.W. (2020). Levonorgestrel-releasing intrauterine system versus endometrial ablation for heavy menstrual bleeding. Am. J. Obstet. Gynecol..

[12] Munro M.G., Critchley H.O., Broder M.S., Fraser I.S., FIGO Working Group on Menstrual Disorders (2011). FIGO classification system (PALM-COEIN) for causes of abnormal uterine bleeding in nongravid women of reproductive age. Int. J. Gynecol. Obstet..

[13] Shubham D., Kawthalkar A.S. (2017). Critical evaluation of the PALM-COEIN classification system among women with abnormal uterine bleeding in low-resource settings. Int. J. Gynecol. Obstet..

[14] Abis P., Bigozzi M.A., Dotto J., Petriglia C., Neri M., Cornacchia S., Angioni S., Loddo A. (2020). Pain Management During Office Hysteroscopy: A Survey of Hysteroscopists. Surg. Technol. Int..

[15] Litta P., Conte L., De Marchi F., Saccardi C., Angioni S. (2013). Pregnancy outcome after hysteroscopic myomectomy. Gynecol. Endocrinol..

[16] Litta P., Leggieri C., Conte L., Dalla Toffola A., Multinu F., Angioni S. (2014). Monopolar versus bipolar device: Safety, feasibility, limits and perioperative complications in performing hysteroscopic myomectomy. Clin. Exp. Obstet. Gynecol..

[17] Vitale S.G., Haimovich S., Riemma G., Ludwin A., Zizolfi B., De Angelis M.C., Carugno J. (2020). Innovations in hysteroscopic surgery: Expanding the meaning of “in-office”. Minim. Invasive Ther. Allied Technol..

[18] Kumar V., Chodankar R., Gupta J.K. (2016). Endometrial Ablation for Heavy Menstrual Bleeding. Women’s Health.

[19] Vitale S.G., Laganà A.S., Caruso S., Garzon S., Vecchio G.M., La Rosa V.L., Casarin J., Ghezzi F. (2021). Comparison of three biopsy forceps for hysteroscopic endometrial biopsy in postmenopausal patients (HYGREB-1): A multicenter, single-blind randomized clinical trial. Int. J. Gynaecol. Obstet..

[20] Riemma G., Vitale S.G., Manchanda R., Rathore A., Török P., De Angelis C., Urman B., Sareri M.I., La Verde M., Carugno J. (2022). The role of hysteroscopy in reproductive surgery: Today and tomorrow. J. Gynecol. Obstet. Hum. Reprod..

[21] Gao W., Zhang L., Li W., Li J., Wang W., Zhao W., Feng L. (2012). Three-year follow-up results of polypectomy with endometrial ablation in the management of endometrial polyps associated with tamoxifen in Chinese women. Eur. J. Obstet. Gynecol. Reprod. Biol..

[22] Moher D., Liberati A., Tetzlaff J., Altman D.G., The PRISMA Group (2009). Preferred Reporting Items for Systematic Reviews and Meta-Analyses: The PRISMA Statement. J. Clin. Epidemiol..

[23] Higgins J.P.T. (2020). Cochrane Collaboration. Cochrane Handbook for Systematic Reviews of Interventions.

[24] McPherson A., Martin C.R. (2012). A review of the measurement properties of the 36-item short-form health survey (SF-36) to determine its suitability for use in an alcohol-dependent population. J. Psychiatr. Ment. Health Nurs..

[25] Yamamoto-Furusho J.K., Sarmiento-Aguilar A., García-Alanis M., Gómez-García L.E., Toledo-Mauriño J., Olivares-Guzmán L., Fresán-Orellana A. (2018). Hospital Anxiety and Depression Scale (HADS): Validation in Mexican patients with inflammatory bowel disease. Gastroenterol. Hepatol..

[26] Bhattacharya S., Cameron I.M., Parkin D.E., Abramovich D.R., Mollison J., Pinion S.B., Alexander D.A., Grant A., Kitchener H.C. (1997). A pragmatic randomised comparison of transcervical resection of the endometrium with endometrial laser ablation for the treatment of menorrhagia. Br. J. Obstet. Gynaecol..

[27] Pinion S.B., Parkin D.E., Abramovich D.R., Naji A., Alexander D.A., Russell I.T., Kitchener H.C. (1994). Randomised trial of hysterectomy, endometrial laser ablation, and transcervical endometrial resection for dysfunctional uterine bleeding. BMJ.

[28] Alexander D.A., Naji A.A., Pinion S.B., Mollison J., Kitchener H.C., Parkin D.E., Abramovich D.R., Russell I.T. (1996). Randomised trial comparing hysterectomy with endometrial ablation for dysfunctional uterine bleeding: Psychiatric and psychosocial aspects. BMJ.

[29] Brun J.-L., Raynal J., Burlet G., Galand B., Quéreux C., Bernard P. (2006). Cavaterm thermal balloon endometrial ablation versus hysteroscopic endometrial resection to treat menorrhagia: The French, multicenter, randomized study. J. Minim. Invasive Gynecol..

[30] Crosignani P.G., Vercellini P., Apolone G., De Giorgi O., Cortesi I., Meschia M. (1997). Endometrial resection versus vaginal hysterectomy for menorrhagia: Long-term clinical and quality-of-life outcomes. Am. J. Obstet. Gynecol..

[31] Dwyer N., Hutton J., Stirrat G.M. (1993). Randomised controlled trial comparing endometrial resection with abdominal hysterectomy for the surgical treatment of menorrhagia. BJOG Int. J. Obstet. Gynaecol..

[32] Loffer F.D., Grainger D. (2002). Five-Year Follow-up of Patients Participating in a Randomized Trial of Uterine Balloon Therapy versus Rollerball Ablation for Treatment of Menorrhagia. J. Am. Assoc. Gynecol. Laparosc..

[33] Zupi E., Zullo F., Marconi D., Sbracia M., Pellicano M., Solima E., Sorrenti G. (2003). Hysteroscopic endometrial resection versus laparoscopic supracervical hysterectomy for menorrhagia: A prospective randomized trial. Am. J. Obstet. Gynecol..

[34] Zupi E., Centini G., Lazzeri L., Finco A., Zullo F., Exacoustos C. (2015). Hysteroscopic Endometrial Resection Versus Laparoscopic Supracervical Hysterectomy for Abnormal Uterine Bleeding: Long Term Follow-Up of a Prospective Randomized Trial. J. Minim. Invasive Gynecol..

[35] Meyer W.R., Walsh B.W., Grainger D.A., Peacock L.M., Loffer F.D., Steege J.F. (1998). Thermal Balloon and Rollerball Ablation to Treat Menorrhagia: A Multicenter Comparison. Obstet. Gynecol..

[36] Sculpher M.J., Dwyer N., Byford S., Stirrat G.M. (1996). Randomised trial comparing hysterectomy and transcervical endometrial resection: Effect on health related quality of life and costs two years after surgery. BJOG Int. J. Obstet. Gynaecol..

[37] Cooper K., Breeman S., Scott N., Scotland G., Clark J., Hawe J., Hawthorn R., Phillips K., MacLennan G., Wileman S. (2019). Laparoscopic supracervical hysterectomy versus endometrial ablation for women with heavy menstrual bleeding (HEALTH): A parallel-group, open-label, randomised controlled trial. Lancet.

[38] Marjoribanks J., Lethaby A., Farquhar C., Farquhar C. (2016). Surgery versus medical therapy for heavy menstrual bleeding. Cochrane Database Syst. Rev..

[39] Fergusson R.J., Lethaby A., Shepperd S., Farquhar C. (2013). Endometrial resection and ablation versus hysterectomy for heavy menstrual bleeding. Cochrane Database Syst. Rev..

[40] Sharp H.T. (2006). Assessment of New Technology in the Treatment of Idiopathic Menorrhagia and Uterine Leiomyomata. Obstet. Gynecol..

[41] Herman M.C., Penninx J.P.M., Mol B.W., Bongers M.Y. (2013). Ten-year follow-up of a randomised controlled trial comparing bipolar endometrial ablation with balloon ablation for heavy menstrual bleeding. BJOG Int. J. Obstet. Gynaecol..

[42] Gurtcheff S.E., Sharp H.T. (2004). Complications Associated With Global Endometrial Ablation: The Utility of the MAUDE Database. Obstet. Gynecol..

[43] Soini T., Rantanen M., Paavonen J., Grénman S., Mäenpää J., Pukkala E., Gissler M., Hurskainen R. (2017). Long-term Follow-up After Endometrial Ablation in Finland: Cancer Risks and Later Hysterectomies. Obstet. Gynecol..

[44] Wishall K.M., Price J., Pereira N., Butts S.M., Della Badia C.R. (2014). Postablation Risk Factors for Pain and Subsequent Hysterectomy. Obstet. Gynecol..

[45] Vitale S.G., Riemma G., Carugno J., Chiofalo B., Vilos G.A., Cianci S., Budak M.S., Lasmar B.P., Raffone A., Kahramanoglu I. (2020). Hysteroscopy in the management of endometrial hyperplasia and cancer in reproductive aged women: New developments and current perspectives. Transl. Cancer Res..

[46] De Franciscis P., Riemma G., Schiattarella A., Cobellis L., Guadagno M., Vitale S.G., Mosca L., Cianci A., Colacurci N. (2019). Concordance between the Hysteroscopic Diagnosis of Endometrial Hyperplasia and Histopathological Examination. Diagnostics.

[47] Liu L., Wang T., Lei B. (2021). High-intensity focused ultrasound (HIFU) ablation versus surgical interventions for the treatment of symptomatic uterine fibroids: A meta-analysis. Eur. Radiol..

[48] Toub D.B. (2017). A New Paradigm for Uterine Fibroid Treatment: Transcervical, Intrauterine Sonography-Guided Radiofrequency Ablation of Uterine Fibroids with the Sonata System. Curr. Obstet. Gynecol. Rep..

[49] Manyonda I., Belli A.-M., Lumsden M.-A., Moss J., McKinnon W., Middleton L.J., Cheed V., Wu O., Sirkeci F., Daniels J.P. (2020). Uterine-Artery Embolization or Myomectomy for Uterine Fibroids. N. Engl. J. Med..

[50] Angioni S., Pontis A., Nappi L., Sedda F., Sorrentino F., Litta P., Haimovich S., Melis G.B. (2016). Endometrial ablation: First- vs. second-generation techniques. Minerva Ginecol..

[51] Litta P., Nappi L., Florio P., Mencaglia L., Franchini M., Angioni S. (2014). Proposal of a modified transcervical endometrial resection (TCER) technique for menorrhagia treatment. Feasibility, efficacy, and patients’ acceptability. Gynecol. Surg..

