# Supplementary material for: Quality of Life, Anxiety and Depression in Women Treated with Hysteroscopic Endometrial Resection or Ablation for Heavy Menstrual Bleeding: Systematic Review and Meta-Analysis of Randomized Controlled Trials
Source: Medicina (Kaunas). 2022 Nov 17;58(11):1664. doi: 10.3390/medicina58111664 (PMC9695759; doi:10.3390/medicina58111664)
Supplement: Supplementary file 1 [file medicina-58-01664-s001.zip › medicina-1958690-supplementary.pdf]

Table S1. Search query for MEDLINE (accessed through PubMed=

(((("endometrial"[All Fields] AND ("resect"[All Fields] OR "resectability"[All Fields] OR "resectable"[All Fields] OR "resectates"[All Fields] OR "resected"[All Fields] OR "resecting"[All Fields] OR "resection"[All Fields] OR "resectional"[All Fields] OR "resectioned"[All Fields] OR "resectioning"[All Fields] OR "resections"[All Fields] OR "resective"[All Fields] OR "resects"[All Fields])) OR ("endometrial ablation techniques"[MeSH Terms] OR ("endometrial"[All Fields] AND "ablation"[All Fields] AND "techniques"[All Fields]) OR "endometrial ablation techniques"[All Fields] OR ("endometrial"[All Fields] AND "ablation"[All Fields]) OR "endometrial ablation"[All Fields])) AND ("hysteroscopy"[MeSH Terms] OR "hysteroscopy"[All Fields] OR "hysteroscopies"[All Fields] OR "resectoscopy"[All Fields])) AND ("hysterectomy"[MeSH Terms] OR "hysterectomy"[All Fields] OR "hysterectomies"[All Fields])) AND (randomizedcontrolledtrial[Filter])
